# Comparison of initial and tertiary centre second opinion reads of multiparametric magnetic resonance imaging of the prostate prior to repeat biopsy

**DOI:** 10.1007/s00330-016-4635-5

**Published:** 2016-10-24

**Authors:** Nienke L. Hansen, Brendan C. Koo, Ferdia A. Gallagher, Anne Y. Warren, Andrew Doble, Vincent Gnanapragasam, Ola Bratt, Christof Kastner, Tristan Barrett

**Affiliations:** 1 Department of Diagnostic and Interventional Radiology, University Hospital RWTH Aachen, Pauwelsstr. 30, 52074 Aachen, Germany; 2 CamPARI Clinic, Addenbrooke’s Hospital and University of Cambridge, Hills Road, CB2 0QQ Cambridge, UK; 3 Department of Radiology, Addenbrooke’s Hospital and University of Cambridge, Hills Road, CB2 0QQ Cambridge, UK; 4 Department of Pathology, Addenbrooke’s Hospital, Hills Road, CB2 0QQ Cambridge, UK; 5 Department of Urology, Addenbrooke’s Hospital, Hills Road, CB2 0QQ Cambridge, UK; 6 Department of Radiology, University of Cambridge School of Clinical Medicine, Box 218, Cambridge Biomedical Campus, Cambridge, CB2 0QQ UK

**Keywords:** Magnetic resonance imaging, Second read, Prostate cancer, Transperineal prostate biopsy, MR/ultrasound fusion biopsy

## Abstract

**Objectives:**

To investigate the value of second-opinion evaluation of multiparametric prostate magnetic resonance imaging (MRI) by subspecialised uroradiologists at a tertiary centre for the detection of significant cancer in transperineal fusion prostate biopsy.

**Methods:**

Evaluation of prospectively acquired initial and second-opinion radiology reports of 158 patients who underwent MRI at regional hospitals prior to transperineal MR/untrasound fusion biopsy at a tertiary referral centre over a 3-year period. Gleason score (GS) 7-10 cancer, positive predictive value (PPV) and negative (NPV) predictive value (±95 % confidence intervals) were calculated and compared by Fisher’s exact test.

**Results:**

Disagreement between initial and tertiary centre second-opinion reports was observed in 54 % of cases (86/158). MRIs had a higher NPV for GS 7-10 in tertiary centre reads compared to initial reports (0.89 ± 0.08 vs 0.72 ± 0.16; *p* = 0.04), and a higher PPV in the target area for all cancer (0.61 ± 0.12 vs 0.28 ± 0.10; *p* = 0.01) and GS 7-10 cancer (0.43 ± 0.12 vs 0.2 3 ± 0.09; *p* = 0.02). For equivocal suspicion, the PPV for GS 7-10 was 0.12 ± 0.11 for tertiary centre and 0.11 ± 0.09 for initial reads; *p* = 1.00.

**Conclusions:**

Second readings of prostate MRI by subspecialised uroradiologists at a tertiary centre significantly improved both NPV and PPV. Reporter experience may help to reduce overcalling and avoid overtargeting of lesions.

***Key Points*:**

• *Multiparametric MRIs were more often called negative in subspecialist reads (41 % vs 20 %)*.

• *Second readings of prostate mpMRIs by subspecialist uroradiologists significantly improved NPV and PPV*.

• *Reporter experience may reduce overcalling and avoid overtargeting of lesions*.

• *Greater education and training of radiologists in prostate MRI interpretation is advised*.

**Electronic supplementary material:**

The online version of this article (doi:10.1007/s00330-016-4635-5) contains supplementary material, which is available to authorized users.

## Introduction

Multiparametric magnetic resonance imaging (mpMRI) of the prostate is increasingly used to identify men with significant prostate cancer [1]. It has been shown to be effective for the detection and local staging of prostate cancers and can preferentially detect clinically relevant index tumours of higher grade and a size >5 mm [2]. A normal mpMRI has been shown to have a high negative predictive value (NPV) (90-98 %) for the presence of clinically significant disease in biopsy [3–5]. However, these literature-reported rates are typically from tertiary referral centres with optimised MRI protocols and subspecialist reporters. Even with expert reads, it is estimated that the use of mpMRI may lead to as many as 25 % of significant cancers being missed, when a radical prostatectomy specimen is used as the reference method [6, 7]. One reason for inaccurate detection of significant cancer in mpMRI could be that mpMRI interpretation accuracy highly depends on the experience of the reader [8–14]. Currently, it is estimated that 100 mpMRI reports supervised by a systematic double-reader and validated by histopathology are needed to gain sufficient reader competence [15], and subsequently at least 50 mpMRIs per year are required to maintain experience levels [16]. Whilst PI-RADS version 1 focused mainly on minimal and optimal MRI protocol standards, the more recently updated PI-RADS version 2 concentrates on standardisation of reading, highlighting a perceived problem [17, 18].

In our region, patients with previous negative biopsies or on active surveillance are often referred from local hospitals to our tertiary centre for further biopsies. These patients have usually undergone previous mpMRI at their referring hospital, which is then second-read by local subspecialist uroradiologists at the tertiary centre prior to biopsy. It has previously been shown that second-opinion interpretations of mpMRI significantly improve sensitivity for extracapsular extension of prostate cancer, even after adjustment for differences in imaging techniques [13], with limited retrospective data also suggesting this may be the case for improving detection of clinically relevant lesions in false-negative mpMRIs [19]. Thus, the aim of this study was to investigate whether prospective second opinion evaluation of prostate mpMRI by subspecialist uroradiologists at a tertiary centre affects the predictive values of the report prior to transperineal fusion biopsy.

## Materials and methods

### Study population

This single-institution study was part of an evaluation of transperineal prostate biopsies with the need for informed consent for data analysis waived by the local ethics committee. From April 2013 to February 2016, 158 consecutive patients referred for transperineal prostate biopsies from regional hospitals and meeting inclusion criteria were included for analysis. Minimum MRI criteria were based on the European Society of Urogenital Radiology (ESUR) 2012 guidance (below) [20]. Patients were referred through an agreed pathway either for active surveillance for repeat surveillance biopsy, or in patients with negative systematic biopsy but ongoing suspicion of cancer based on prostate-specific antigen (PSA), symptoms or prior high risk biopsy, based on UK guidance. The clinical objective was to identify significant cancer necessitating treatment, defined as Gleason score 7-10. Thirty-three patients on active surveillance for Gleason 3+3 disease were included for retrospective analysis; 125 patients had previous negative systematic transrectal ultrasound (TRUS)-guided biopsies (Supplementary Fig. 1). Patients on active monitoring for Gleason score 7 cancer were therefore excluded from the analysis, as their disease already met our criteria for clinically significant cancer. The Standards of Reporting for MRI-targeted Biopsy Studies (START) were used to describe the study population, the conduct and reporting of the MRI, and the conduct of the biopsy and the Standards of Reporting of Diagnostic Accuracy (STARD) were used to describe and discuss the results [21, 22].

### Magnetic resonance imaging

Patients underwent prostate mpMRI at seven different regional hospitals on either 1.5-T (87/158) or 3.0-T (71/158) MRI scanners with surface coil and no endorectal coil. Minimum sequence requirements for inclusion in the study were axial T_1_-weighted images (T1WIs) of the pelvis, high resolution axial T_2_-weighted images (T2WIs) of the prostate and diffusion-weighted imaging (DWI), with a minimum of two *b* values, with a high *b* value of 800-1,000 [20]. Apparent diffusion coefficient (ADC) maps were calculated for all patients. Eight percent (12/158) of mpMRIs included dynamic contrast-enhanced sequences.

### Image analysis

All mpMRIs were performed and first-read at seven regional referral centres by 28 different radiologists. Data on the referring radiologists’ experience in reporting mpMRIs of the prostate were not available. All mpMRI images were prospectively second-read at our own tertiary centre, with external reports available. Second reads were performed by one of two subspecialist uroradiologists with 6 (over 1,500 cases) and 4 years (over 1,000 cases) of experience in reading prostate MRI. In the second-read, T2WI and DWI sequences were evaluated using a Likert scale of tumour probability, based on the Prostate Imaging Reporting and Data System (PI-RADS version 1) structured scoring criteria developed by the ESUR [20] and a final score was defined by combining all scores for T2WI and DWI sequences as recommended in PI-RADS version 2 [23]. This second read was performed contemporaneously, soon after initial MRI acquisition and before the biopsy procedure, therefore prospectively affecting the biopsy targeting. For patients in whom more than one MRI had been performed, all studies were available to both internal and external readers. The Likert-based scoring system was as follows: 1 = cancer highly unlikely, 2 = cancer unlikely, 3 = equivocal for cancer, 4 = cancer likely, 5 = cancer highly likely. Lesion probability only, rather than quantifiable measures such as ADC value and measurements were recorded. In cases where external reports did not explicitly state a PIRADS/Likert score or location, the reports were reviewed by two research radiologists in consensus, blinded to the clinical details and retrospectively grouped for suspicion of cancer as no suspicion (Likert 1-2), equivocal (Likert 3) or suspicion (Likert 4-5) for cancer.

### Biopsy

The Biopsee^TM^ transperineal MRI/TRUS fusion biopsy system version 1 or 2 (Medcom, Darmstadt, Germany) was used for all biopsies. All patients had 18-24 systematic biopsies taken according to the Ginsburg protocol, using a spring-loaded biopsy gun with an 18-gauge needle [5, 24]. In patients with MRI lesions prospectively called by the subspecialist reader, two biopsy cores were taken from each Likert 3-5 lesion before the systematic biopsies. In the systematic biopsy, two biopsy cores were sampled from each of 12 sectors, starting with the anterior sectors. All procedures were undertaken by one of three urologists with several years’ experience of transperineal biopsy using the Biopsee MRI/TRUS fusion biopsy system.

### Histopathology

All biopsies were reported by a specialist uropathologist and were reviewed a second time, by another uropathologist, prior to discussion at a multidisciplinary team meeting. Biopsies were reviewed according to the ISUP 2005 recommendations [25]. The final Gleason score was used as data for this study.

### Statistics

The absolute and relative agreements of initial and tertiary centre reads were calculated. Kappa coefficient calculation was used to compare distribution into the different probability subgroups. It was also analysed in how many cases lesions were called in both reads, how many reports called different lesions and how often there was no corresponding lesion at all with the other read being rated as non-suspicious.

Benign histopathology, prostatitis and Gleason score (GS) 3 + 3 = 6 were considered as negative histopathology. GS 7-10 cancer detection rate, all cancer detection rate, and positive (PPV) and negative (NPV) predictive values were calculated for each probability group and both readings, including targeted and systematic biopsy cores in the area that the index lesion was located in. For example: if an index lesion was called in the right anterior, the results for the targeted cores and the systematic cores in the right anterior were used for analysis. If the index lesion was called in the initial read but not targeted after the second read, the systematic cores in the respective area were used for analysis. Fisher’s exact test was used to test for statistically significant difference of cancer proportions.

## Results

Initial reports called 47 % (59/126) index lesions in the transition zone and 53 % (67/126) in the peripheral zone. More than one lesion was called in 36/126 patients in the initial report. After second reading, 44 % (41/94) index lesions were called in the transition zone and 56 % (53/94) in the peripheral zone. More than one lesion was called in 19/94 patients in the subspecialist tertiary-centre report. At transperineal biopsy, 51 % (80/158) of patients had a GS 6-10 prostate cancer (PCa), 30 % (47/158) of patients had a GS ≥3 + 4 PCa and 5 % (8/158) a GS ≥4 + 3 PCa. The clinical characteristics are shown in Table 1.

**Table 1:** Clinical characteristics of the patients included in the study

	Total *n*	IQR
Median age (years)	65	59-69
Median PSA (ng/mL)	7.7	5.8-12.7
Median volume (cc)	59	40-78
Median PSA density (ng/mL/cm^3^)	0.14	0.09-0.22
Median number of target cores	2	2-4
Median numbers of systematic cores	24	24-24

*PSA* prostate-specific antigen, *IQR* interquartile range

### Agreement of probability scoring

The strength of agreement between initial reports and tertiary centre second-reads was poor in all three groups (Table 2). Overall, 135 index lesions were identified in 158 patients by either one or both reads, 126 in initial reports and 94 in tertiary-centre second reads. In 33 % (45/135) the same lesion was called in both reads, in 27 % (37/135) different lesions were called and in 39 % (53/135) there was no corresponding lesion at all with the other read being rated as non-suspicious.

**Table 2:** Cross-table of probability scoring between initial reports and second reads. There were 46 % (72/158; *kappa* value = 0.177) agreements in grouping for suspicion of cancer as no suspicion (Likert 1-2), equivocal (Likert 3) and suspicion (Likert 4-5) for cancer. The strength of agreement into the broad groups of MRI being either negative (Likert 1-2) or suspicious (Likert 3-5) was fair (106/158; *kappa* value = 0.258)

	Subspecialist second read							
Initial read	Likert 1-2 *n* (%)	(%)	Likert 3 *n*	(%)	Likert 4-5 *n*	(%)	Total *n*	(%)
Likert 1-2	*22*	*14 %*	3	2 %	7	4 %	32	20 %
Likert 3	18	11 %	*12*	*8 %*	16	10 %	46	29 %
Likert 4-5	24	15 %	18	11 %	*38*	*24 %*	80	51 %
Total	64	41 %	33	21 %	61	39 %	158	100 %

### NPV of negative MRI (Likert 1-2)

**Fig. 1:**
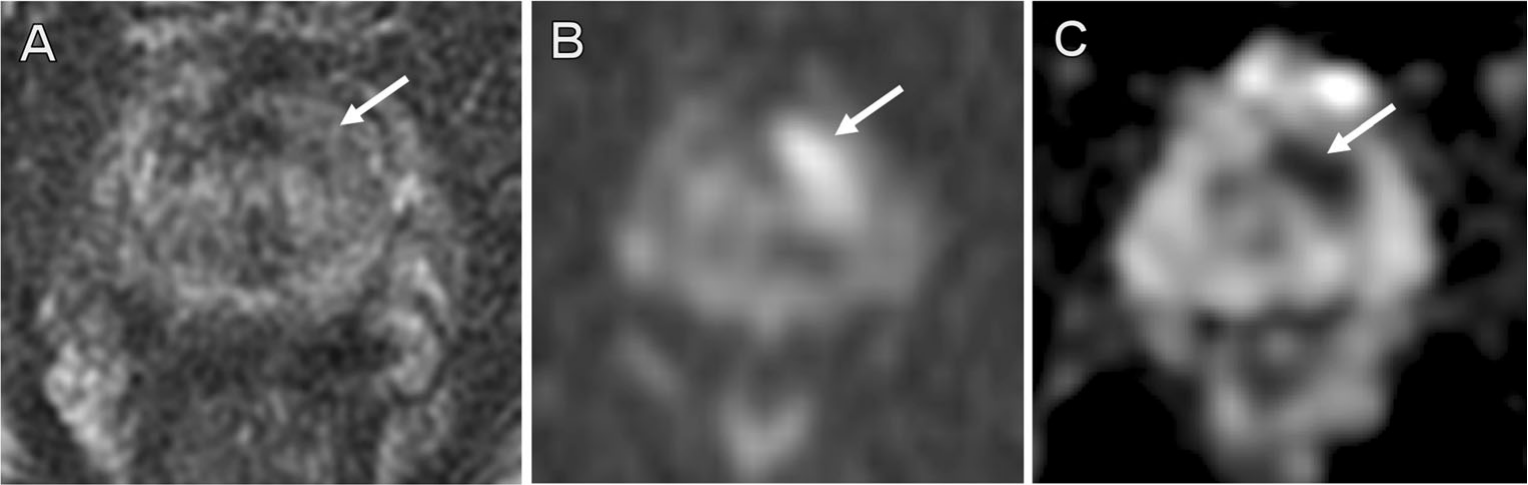
False-negative external report. A 70-year-old patient with 10.4 ng/ml PSA and previous negative TRUS biopsy. MRI reported as negative externally. Second report identified a high probability (PIRADS-5) 15-mm target in the left anterior apex transition zone (*arrows*), with homogeneous low T2-signal (**a**), and restricted diffusion on *b*-1,400 imaging (**b**) and ADC map (**c**). Transperineal biopsy found Gleason 3 + 3 disease in 30 % of both target cores

Multiparametric MRIs were more often called negative in tertiary-centre reads than in initial reports (41 % vs 20 % Likert 1-2; *p* = 0.0001) (Table 3). The corresponding NPV for GS ≥3 + 4 cancer was significantly higher for tertiary-centre second reads (0.89; 57/64) compared to initial reports (0.72; 23/32) despite the more frequent calling by tertiary-centre readers; *p* = 0.044 (Fig. 1). NPVs for higher grade (GS ≥4 + 3) tumours were also significantly higher at 0.97 (62/64) and 0.84 (27/32), respectively; *p* = 0.039.

**Table 3:** Negative predictive value of non-suspicious mpMRI (Likert 1-2) after a transperineal MRI/TRUS-fusion guided targeted and 18–24-core systematic prostate biopsy according to the Ginsburg protocol

Likert 1-2	Total (*n*)	% of total	GS 7-10 (*n*)	NPV	95 % CI	*p* value	GS ≥4 + 3 (*n*)	NPV	95 % CI	*p* value
External report	32	20 %	9	0.72	0.56-0.88	0.04	5	0.84	0.71-0.97	0.04
Subspecialist	64	41 %	7	0.89	0.81-0.97		2	0.97	0.93-1.01	

*GS* Gleason score, *NPV* negative predictive value, *CI* confidence interval

### PPV of equivocal mpMRI (Likert 3)

Regarding the outcome for the individual target area, PPVs were not significantly different between tertiary centre and initial reads with 0.24 (8/33) and 0.20 (9/46) for any cancer, respectively; *p* = 0.782; and 0.12 (4/33) and 0.11 (5/46) for GS7-10; *p* = 1.000 (Table 4).

**Table 4:** The positive predictive values of equivocal multiparametric MRI (Likert 3) using a transperineal MRI/TRUS-fusion guided targeted and 18–24-core systematic prostate biopsy as the reference test

Likert 3	Total (*n*)	% of total	GS 6-10 (*n*)	PPV	95 % CI	*p* value	GS 7-10 (*n*)	PPV	95 % CI	*p* value
*Target area only*										
External report	46	29 %	9	0.20	0.08-0.32	0.78	5	0.11	0.02-0.20	1.00
Subspecialist	33	21 %	8	0.24	0.09-0.39		4	0.12	0.01-0.23	
*Total biopsy*										
External report	46	29 %	22	0.48	0.33-0.62	1.00	11	0.24	0.12-0.36	0.59
Subspecialist	33	21 %	15	0.45	0.28-0.62		6	0.18	0.05-0.31	

*GS* Gleason score, *PPV* positive predictive value, *CI* confidence interval

### PPV of suspicious mpMRI (Likert 4-5)

**Fig. 2:**
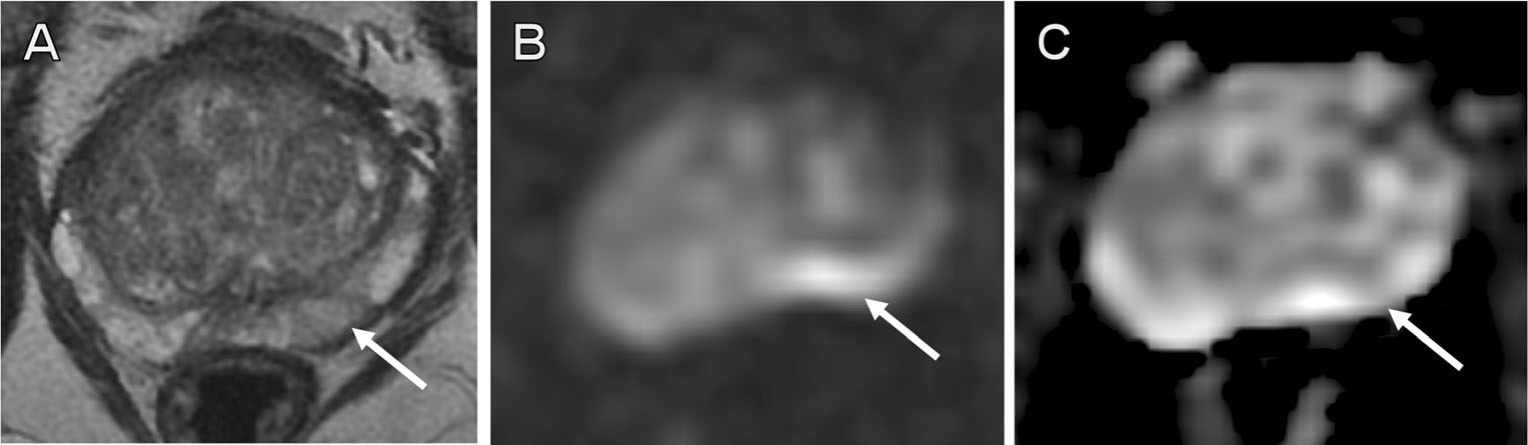
False-positive external report. A 64-year-old patient with 7.1 ng/ml PSA and previous negative TRUS biopsy. External report described a high probability target in the left mid peripheral zone. Second read called a negative MRI, with linear areas of intermediate T2 signal in the left mid (**a**, PIRADS-2) and high signal on *b*-1,400 imaging (**b**) thought to be artefactual due to rectal gas, and without convincing low signal on ADC maps (**c**). Subsequent transperineal template biopsy showed all 24 cores to be benign

**Fig. 3:**
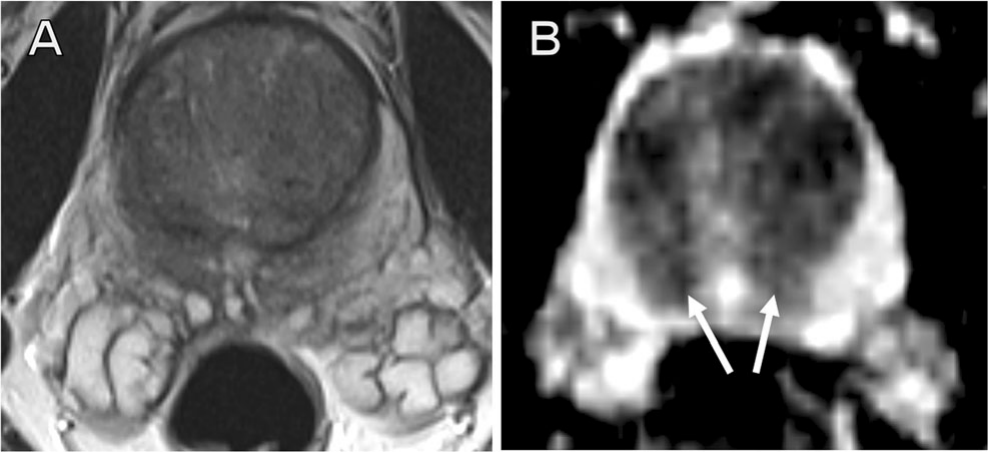
False-positive external report. A 50-year-old patient with16.6 ng/ml PSA and previous negative TRUS biopsy. External report described high probability targets bilaterally and medially at the base peripheral zone. Second read called a negative MRI, with normal central zone demonstrating low T2 signal (**a**) and low signal on ADC maps (**b**, *arrows*). Subsequent transperineal template biopsy showed all 24 cores to be benign

Multiparametric MRIs were less often called suspicious in tertiary-centre reads than in initial reports (39 % vs 51 % Likert 4-5; *p* = 0.04) (Table 5). Regarding the outcome for the individual target area, PPVs for detecting any cancer were significantly higher for tertiary-centre reads (0.61; 37/61) than for initial reads (0.28; 22/80); *p* = 0.0001 (Figs. 2 and 3). For detection of GS 7-10 cancer in the target area, PPVs were 0.43 (26/61) with subspecialist second reads versus 0.23 (18/80) for initial reports; *p* = 0.017. Regarding the final histopathology result for the entire prostate biopsy, PPV for GS7-10 cancer was significantly higher for tertiary-centre second reads (0.56; 34/61) than for to initial reports (0.34; 27/80), despite the less frequent calling by tertiary-centre readers; *p* = 0.011.

**Table 5:** The positive predictive values of suspicious multiparametric MRI (Likert 4-5) using a transperineal MRI/TRUS-fusion guided targeted and 18–24-core systematic prostate biopsy as the reference test

Likert 4-5	Total (n)	% of total	GS 6-10 (n)	PPV	95 % CI	*p* value	GS 7-10 (n)	PPV	95 % CI	*p* value
*Target area only*										
External report	80	51 %	22	0.28	0.18-0.38	0.01	18	0.23	0.14-0.32	0.02
Subspecialist	61	39 %	37	0.61	0.49-0.73		26	0.43	0.31-0.55	
*Total biopsy*										
External report	80	51 %	44	0.55	0.44-0.66	0.08	27	0.34	0.24-0.44	0.01
Subspecialist	61	39 %	43	0.70	0.59-0.82		34	0.56	0.44-0.68	

*GS* Gleason score, *PPV* positive predictive value, *CI* confidence interval

## Discussion

Our study shows that second-opinion readings of mpMRI by subspecialised uroradiologists at a tertiary centre significantly improved diagnostic accuracy. Multiparametric MRIs were more often called negative in tertiary-centre reads than in initial reports (41 % vs 20 % Likert 1-2). A negative mpMRI tertiary-centre second read by subspecialists had a significantly higher NPV for cancer GS 7-10 than for initial regional reports (0.89 vs 0.72). PPVs of equivocal MRIs for detecting cancer in the target area were not significantly different for the initial and tertiary centre readers with 0.11 vs 0.12 (*p* = 1.00), although of note, these were called less frequently called by the latter. Multiparametric MRIs were also less often called suspicious in tertiary-centre reads than in initial reports with 39 % vs 51 % (*p* = 0.04). A suspicious mpMRI by tertiary-centre read had a significantly higher PPV both for detecting any cancer (0.61 vs 0.28) and for detecting GS 7-10 cancer (0.43 vs 0.23) in the target area.

Even though negative mpMRIs were reported more frequently by tertiary-centre radiologists, the NPV of 0.89 was equivalent to that reported in existing literature [2, 3, 5, 26], with a significantly lower NPV of 0.72 observed for initial reads. This is particularly relevant with the increasing use of mpMRI prior to initial biopsy, with retrospective data on its high NPV leading to prospective studies investigating whether a negative MRI can safely allow men to avoid a biopsy and its associated harms [27]. Our results support the findings of Gaziev et al. [14], who reported that radiologists undergo a learning curve with NPVs increasing from 0.67 to 0.89 for excluding significant cancer with increasing experience. Additionally, avoiding biopsies in men with a negative MRI according to tertiary-centre uroradiologists would have led to missing only 3 % GS ≥4 + 3 PCa, compared with 16 % if an external radiologist had assigned the MRI as negative. Urologists need to be aware of this risk when deciding which patients need to undergo re-biopsy in the context of an ongoing clinical suspicion of cancer, or which can be followed-up with active surveillance.

Suspicious mpMRIs were called less frequently than in initial reports, but maintained a higher PPV of 0.61 for detecting any cancer in the target area and 0.56 for detection of GS7-10 PCa in the total prostate biopsy; results which are similar to those previously published in centres with subspecialist uroradiologists [5, 26]. In contrast, the PPV for initial reports to find any cancer in the target area was 0.28. This implies that inexperienced radiologists overcall suspicious lesions, while missing significant cancer elsewhere in the prostate. This is especially relevant for urologists that want to undertake targeted biopsies only, without systematic cores. Reporter experience may reduce overcalling and therefore avoid overtargeting of lesions called by less experienced readers. Gaziev et al. [14] found that the detection rate of targeted biopsy rises from 0.27 up to 0.63 with increasing experience. In our study, PPV was 0.28 for initial readers, which is at the lower end of the learning curve reported by Gaziev et al. Conversely, second reading by subspecialists at a tertiary centre in our study improved the PPV for finding any cancer in the target area to 0.61.

A strength of our study is that we used a combination of targeted and a 24-core systematic transperineal biopsy as the reference. Next to radical prostatectomy specimens and transperineal mapping, this is the most valid means of assessing for clinically significant PCa. Additionally, benign cases will not undergo prostatectomy and therefore data for true negatives are not available on a whole-gland, patient-by-patient basis. Limitations of this study include its retrospective analysis, with potential for allocation bias in review of external reports, and a lack of data on the referring radiologists’ experience in reporting mpMRIs of the prostate. If second reading was negative, the initially called lesions were not targeted in the biopsy. To reduce this potential bias against the initial read, the systematic cores from the equivalent sector were used for analysis. If this study were to be repeated prospectively, all index lesions called by either read could be targeted. Subspecialist readers had the advantage of availability of the initial report, which may bias decision-making. However, alliterative error resulting from the influence of one radiologist on another is more likely to favour concordance [28], and the poor agreement here (44 %) suggests this did not influence decision-making. In addition, we have no information on the proportion of referred patients to our centre and on the referral criteria employed; for instance, cases with concordant clinical assessment and negative imaging may not have been referred to our tertiary centre, which could potentially affect the NPV. However, we feel that our results sufficiently emphasise the necessity of adequate experience for radiologists when reporting prostate mpMRIs.

Our results make the case for subspecialist reading of mpMRI, particularly in the context of repeat biopsy. At our centre, the referral for second reading of studies only represented approximately 10 % of the MRIs performed at the respective centres. As a high-volume institution, we perform more MRIs than these centres, but centralised reporting of all regional studies would still result in a threefold increase in workload. Aside from increasing the number of central subspecialised radiologists, another option may be to provide more training to radiologists at the outside hospitals. A suggested way to achieve this is the adoption of a temporary intermediate competency certification process based on the experience of 50–100 cases with a supervised systematic double-reading by an experienced reader and pathology feedback [15, 16].

## Conclusions

Second reading of prostate mpMRIs by subspecialised uroradiologists at a tertiary centre significantly improved the NPV and PPV. Urologists should be aware that the experience of the reporter will affect the report when making a decision if and how to obtain biopsies.

## Electronic supplementary material

Below is the link to the electronic supplementary material.

Supplementary Fig. 1 Study design flow chart (JPG 6 kb)

High Resolution Image (TIF 167 kb)

## Abbreviations

mpMRI: multiparametric magnetic resonance imaging
PSA: prostate-specific antigen
GS: Gleason score
