# Supplementary figures and images for: Comparison of initial and tertiary centre second opinion reads of multiparametric magnetic resonance imaging of the prostate prior to repeat biopsy
Source: Eur Radiol. 2016 Oct 24;27(6):2259–66. doi: 10.1007/s00330-016-4635-5 (PMC5408042; doi:10.1007/s00330-016-4635-5)

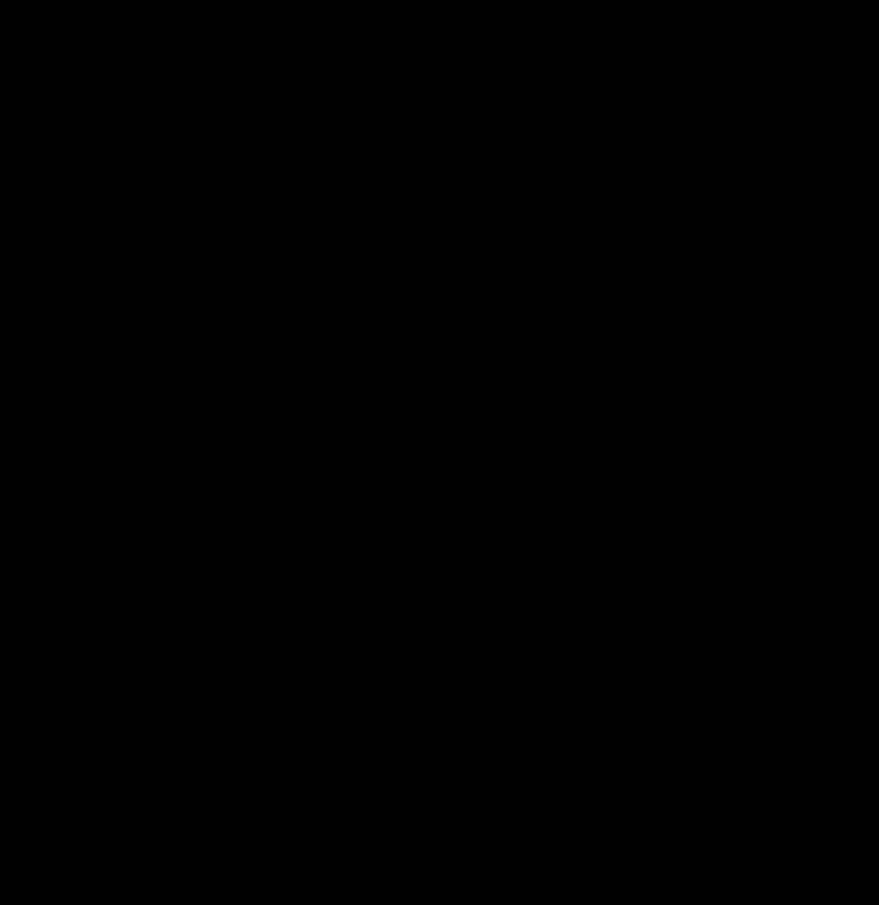

Supplement: Supplementary file 1 — Study design flow chart (JPG 6 kb) [file 330_2016_4635_Fig4_ESM.jpg]

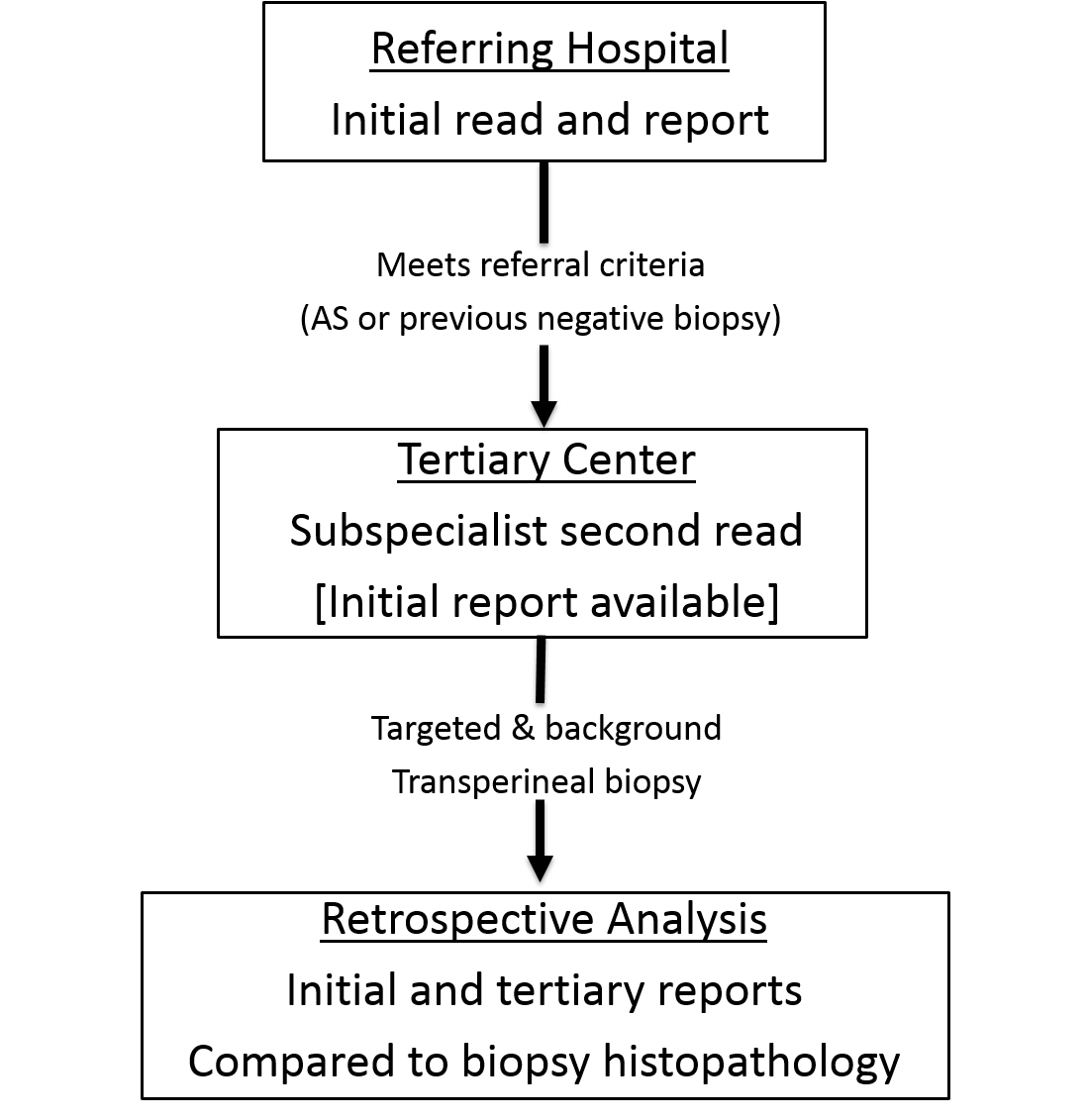

Supplement: Supplementary file 2 — High Resolution Image (TIF 167 kb) [file 330_2016_4635_MOESM1_ESM.tif]
